# Genetic Knock-Down of *Hdac3* Does Not Modify Disease-Related Phenotypes in a Mouse Model of Huntington's Disease

**DOI:** 10.1371/journal.pone.0031080

**Published:** 2012-02-08

**Authors:** Lara Moumné, Ken Campbell, David Howland, Yingbin Ouyang, Gillian P. Bates

**Affiliations:** 1 Department of Medical and Molecular Genetics, King's College London, London, United Kingdom; 2 Taconic, Cranbury, New Jersey, United States of America; 3 CHDI Management/CHDI Foundation, Princeton, New Jersey, United States of America; Emory University, United States of America

## Abstract

Huntington's disease (HD) is an autosomal dominant progressive neurodegenerative disorder caused by an expansion of a CAG/polyglutamine repeat for which there are no disease modifying treatments. In recent years, transcriptional dysregulation has emerged as a pathogenic process that appears early in disease progression and has been recapitulated across multiple HD models. Altered histone acetylation has been proposed to underlie this transcriptional dysregulation and histone deacetylase (HDAC) inhibitors, such as suberoylanilide hydroxamic acid (SAHA), have been shown to improve polyglutamine-dependent phenotypes in numerous HD models. However potent pan-HDAC inhibitors such as SAHA display toxic side-effects. To better understand the mechanism underlying this potential therapeutic benefit and to dissociate the beneficial and toxic effects of SAHA, we set out to identify the specific HDAC(s) involved in this process. For this purpose, we are exploring the effect of the genetic reduction of specific HDACs on HD-related phenotypes in the R6/2 mouse model of HD. The study presented here focuses on HDAC3, which, as a class I HDAC, is one of the preferred targets of SAHA and is directly involved in histone deacetylation. To evaluate a potential benefit of *Hdac3* genetic reduction in R6/2, we generated a mouse carrying a critical deletion in the *Hdac3* gene. We confirmed that the complete knock-out of *Hdac3* is embryonic lethal. To test the effects of HDAC3 inhibition, we used *Hdac3* ^+/−^ heterozygotes to reduce nuclear HDAC3 levels in R6/2 mice. We found that *Hdac3* knock-down does not ameliorate physiological or behavioural phenotypes and has no effect on molecular changes including dysregulated transcripts. We conclude that HDAC3 should not be considered as the major mediator of the beneficial effect induced by SAHA and other HDAC inhibitors in HD.

## Introduction

Huntington's disease (HD) is an autosomal dominant progressive neurodegenerative disorder with a mean age of onset of 40 years. The main clinical manifestations are chorea, cognitive impairment, psychiatric disorders and weight loss. The disease duration is 15–20 years and in the absence of disease modifying treatments, the disease progresses inexorably until death [1]. The mutation responsible for HD is an unstable expansion of a CAG repeat in the *HTT* gene that leads to a polyglutamine expansion in the N-terminus of the huntingtin (HTT) protein [2]. Neuropathologically, HD is characterized by neuronal loss in several brain regions including the striatum and the cortex as well as the deposition of nuclear and cytoplasmic HTT-containing aggregates [3].

A variety of mouse models have been used to study the pathogenic pathways involved in HD [4]. These include the R6/2 model, which is transgenic for a single-copy of exon 1 of human *HTT*, which in our colony carries approximately 200 CAG repeats [5], [6] and the *Hdh*Q150 knock-in model in which 150 CAGs have been inserted into the mouse *Htt* gene [7], [8]. The R6/2 mouse has an early onset progressive phenotype that recapitulates many features of the human disease. Motor and cognitive impairment appear before 6 weeks, HTT aggregation can clearly be detected from 3 weeks, whereas neuronal cell loss in the striatum occurs at later stages [9], [10], [11]. Mice with an average 200 CAG repeats are not usually kept beyond 15 weeks. The early and reproducible phenotype of this mouse line has made it an ideal model screening compounds and performing genetic crosses. At late-stage disease, the R6/2 and *Hdh*Q150 models have developed remarkably similar phenotypes [8], [12], [13], [14] supporting the hypothesis that the generation of a toxic fragment is a critical event in HD pathogenesis [15].

In recent years, transcriptional dysregulation has emerged as a pathogenic process that appears early in disease progression and has been recapitulated across multiple HD model systems including the R6/2 and *Hdh*Q150 mouse models [12], [16]. Histone acetylation/deacetylation is an important mechanism involved in gene transcriptional regulation and altered histone acetylation has been proposed to underlie transcriptional dysregulation in HD [17]. In general, acetylation of lysines by histone acetyl transferases (HAT) leads to activation of transcription whereas removal of acetyl groups by histone deacetylases (HDAC) leads to gene silencing [18]. There are two evolutionary divergent types of HDACs: the zinc-dependent HDACs (human HDAC1-11) and the NAD^+^ dependent HDACs also called sirtuins (human SIRT1-7). Inhibitors of the zinc-dependant HDACs include suberoylanilide hydroxamic acid (SAHA) and trichostatin A (TSA) [19], whereas sirtuins are inhibited by nicotinamide [20]. The human zinc-dependent HDAC family comprises eleven members divided into four classes: I (HDAC1, 2, 3 and 8), IIa (HDAC4, 5, 7 and 9), IIb (HDAC6 and 10) and IV (HDAC11). HDAC inhibition has been shown to improve polyglutamine-dependent phenotypes in several models of HD [21], [22], [23], [24], [25], [26]. Although administration of SAHA improves HD-related phenotypes in the R6/2 mouse model, therapeutic doses have toxic effects and induce profound weight loss [24].

To better understand the mechanism underlying this potential therapeutic benefit and to dissociate the beneficial and toxic effects of SAHA, we set out to identify the specific HDAC(s) involved in this process. For this purpose, we are exploring the effect of the genetic reduction of specific HDACs on HD-related phenotypes in R6/2 mice. Genetic reduction (knock-down or complete knock-out) of *Hdac5, 6, 7 and 9* failed to induce a phenotypic improvement ([27], [28] and unpublished data) whereas knock-down of *Hdac4* induces a significant beneficial effect (unpublished data).

The study presented here focuses on HDAC3, which is the most highly expressed class I HDAC in the brain [29]. This HDAC is of particular interest for several reasons. Class I HDACs are directly involved in histone deacetylation and as a class I HDAC, HDAC3 is one of the main cellular targets of SAHA [30]. A recent study showed that the class I inhibitor HDACi **4b**, which is reported to be more specific for HDAC3 than the other class I HDACs, ameliorated the disease phenotype and reversed many of the transcriptional abnormalities found in the brain of R6/2 mice [26]. Moreover, studies involving genetic reduction of specific HDACs in invertebrate models of HD have implicated class I HDACs in the reduction of polyglutamine-dependent toxicity. In *Drosophila melanosgaster*, knock-down of the class I HDAC *rpd3*, that is homologous to human *HDAC1/2* but also partially homologous to *HDAC3*, suppresses pathogenesis [31]. Similarly, in *C. Elegans*, only the knock-down of the *hda-3*, that shares homology with human class I HDACs, suppressed polyglutamine-dependant toxicity [21]. Finally, the catalytic domain of HDAC4 interacts with HDAC3 via the transcriptional corepressor complex N-CoR/SMRT, and suppression of HDAC4 binding to SMRT/N-CoR and to HDAC3 results in the loss of enzymatic activity associated with HDAC4 [32]. Given the beneficial effect of *Hdac4* knock-down on HD-related phenotypes in R6/2 mice, we might expect that a reduction of *Hdac3* expression would lead a reduced HDAC4 activity and an improvement in R6/2 phenotypes.

To evaluate a potential benefit of *Hdac3* genetic reduction in R6/2, we generated a genetically engineered mouse in which part of the *Hdac3* gene is deleted. We observed that a complete knock-out of *Hdac3* is embryonic lethal. *Hdac3* mRNA levels were reduced to 50% of wild type (WT) in the brains of *Hdac3* ^+/−^ heterozygotes (knock-down) with a corresponding reduction in HDAC3 protein in the nucleus but not in the cytoplasm. We found that *Hdac3* ^+/−^ heterozygotes are viable and fertile, with no overt phenotype. We performed a genetic cross between R6/2 mice and *Hdac3^+/−^* heterozygotes and found that *Hdac3* knock-down does not ameliorate physiological or behavioural phenotypes in R6/2 mice, does not modulate HTT aggregation and has no effect on transcriptional dysregulation. We conclude that HDAC3 should not be considered as the major mediator of the beneficial effect induced by SAHA and other HDAC inhibitors in HD.

## Results

### Conventional heterozygous deletion of *Hdac3*

**Figure 1:**
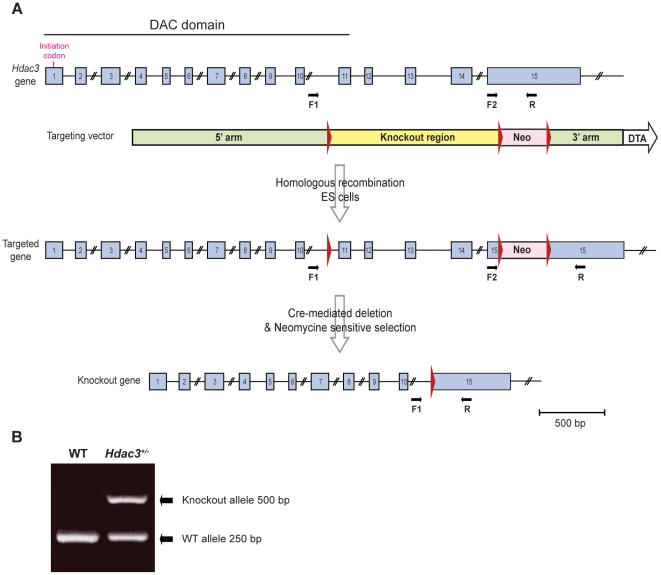
Generation of an *Hdac3* convention knock-out allele. (A) Strategy to generate an *Hdac3* conventional knock-out allele. The genomic structure and the targeting vector are shown. The *Hdac*3 gene contains 15 exons (blue rectangles). LoxP sites (red triangles) were introduced upstream exon 11 and within exon 15. The vector contains a 5′ homology arm covering the exonic and intronic region from intron 3–4 to intron 10–11 and a 3′ homology arm covering a part of exon 15 and the 3′UTR (green rectangle). The conditional knock-out region (yellow rectangle) covers exon 11 to 14 and 5′ end of exon 15. This conditional allele was introduced by homologous recombination in ES cells. The neomycine cassette (pink rectangle) flanked by 2 LoxP sites was removed by electroporation of Cre recombinase in ES cells and the cells containing the allele corresponding to a complete deletion of exon 11 to 14 were selected. Primers used for genotyping are represented as black arrows. F1 = forward 1; F2 = forward 2; R = Reverse. (B) Representative genotyping PCR on mouse genomic DNA. Duplex PCR with F1, F2 and R primers detects both the WT (250 bp band with primers F2/R) and the knock-out (500 bp with primers F1/R) allele.

We generated a conventional null allele of *Hdac3* in order to evaluate whether a reduction in HDAC3 level has beneficial effects in the R6/2 mice. For this purpose, loxP sites were introduced upstream of exon 11 and within exon 15 by homologous recombination inducing a deletion covering exon 11 to 14 and the 5′ end of exon 15 (Fig. 1A). This mutation removes a part of the nuclear localization signal and a C-terminal region necessary for both deacetylase activity and transcriptional repression [33], [34]. Heterozygous F1 mice were generated and deletion of *Hdac3* was confirmed at the genomic level by PCR and sequencing (Fig. 1B). We intercrossed *Hdac3^+/−^* mice in order to generate nullizygotes, but did not obtain any *Hdac3* knock-out offspring at birth or between embryonic stages E8.5 and E15.5. This result reveals that a complete lack of *Hdac3* induces an embryonic lethality prior to E8.5 and confirms previous studies showing that *Hdac3^−/−^* mice die before E9.5 [35], [36]. Given this embryonic lethality we used *Hdac3* ^+/−^ heterozygotes for our study. These mice are viable and fertile, with no overt phenotype.

**Figure 2:**
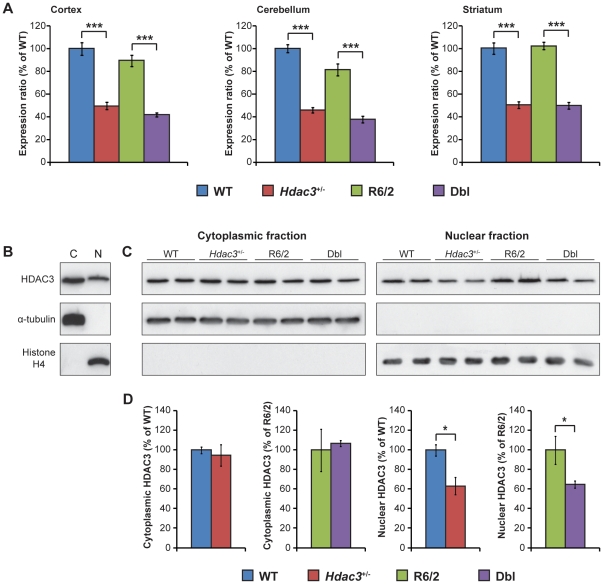
*Hdac3* mRNA and protein expression in *Hdac3+/−* heterozygous mouse brain. (A) *Hdac3* mRNA expression levels in 15 week old mouse cortex, cerebellum and striatum are shown as a relative expression ratio to the WT level. *Hdac3^+/−^* (red) and Dbl (purple) mice express the mRNA at 50% of the WT (blue) and R6/2 (green) levels in all brain regions. Error bars correspond to S.E.M. (n = 8) ***p<0.001. (B) Western blot showing the expression of HDAC3 protein in the cytoplasmic (C) and the nuclear (N) fraction of WT mouse whole brain. Antibodies to α-tubulin (cytoplasmic) and histone H4 (nuclear) were used to control for the purity of the fractions. (C) Representative western blot and (D) quantification of cytoplasmic and nuclear fraction prepared from WT (blue), *Hdac3^+/−^* (red), R6/2 (green) and Dbl (purple) 15 week old-mouse whole brains. Antibodies to α-tubulin (cytoplasmic) and histone H4 (nuclear) were used as both purity and loading controls. Cytoplasmic HDAC3 was not affected by *Hdac3* deletion whereas nuclear HDAC3 was reduced to 60% of the WT level. Error bars correspond S.E.M. (n = 3) *p<0.05.

It was important first to confirm that the *Hdac3* expression level was reduced in *Hdac3^+/−^* mice as it has been previously shown that *Hdac1*, another class I HDAC, autoregulates its expression to wild-type (WT) levels in *Hdac1* ^+/−^ heterozygous mice [37]. It was also important to determine whether *Hdac3* levels might be regulated by the R6/2 transprotein. Therefore, we crossed R6/2 males to *Hdac3* ^+/−^ heterozygous females to generate WT, *Hdac3* ^+/−^, R6/2 and R6/2::*Hdac3* ^+/−^ double mutant (Dbl) mice and performed RT-qPCR on cDNA prepared from cortex, cerebellum and striatum of 15-week old mice. The RT-qPCR primer sequences were located within the deletion (at the junction between exon 14 and 15) so that only the WT allele can be detected. We observed a significant reduction of *Hdac3* mRNA to 50% of the WT level in all the brain regions irrespective of the presence of the R6/2 transprotein (Fig. 2A). In order to verify the specificity of *Hdac3* reduction we analyzed the expression level of the other *Hdacs* by RT-qPCR and showed that these were not changed in *Hdac3^+/−^* mice compared to WT (Fig. S1).

In order to see whether this mRNA reduction translates into a reduction at the protein level, we performed western blotting on whole brain lysates using an HDAC3 specific antibody. Surprisingly, the protein level was only reduced to 80% of the WT level (Fig. S2). As HDAC3 is present in both the nucleus and the cytoplasm in mammalian cells [33], we investigated whether HDAC3 levels might differ between these two intracellular compartments. For this purpose, we performed nuclear and cytoplasmic fractionation of whole brains from 4 week old mice. We first confirmed that HDAC3 is present in both the nucleus and cytoplasm in WT mouse brain (Fig. 2B). Analysis of brains from WT, *Hdac3^+/−^*, R6/2 and Dbl mice revealed that cytoplasmic HDAC3 is not reduced in *Hdac3* ^+/−^ and Dbl brains as compared to WT and R6/2. In contrast, its expression is reduced to 60% of the WT level in *Hdac3* ^+/−^ heterozygotes irrespective of the presence of the R6/2 transgene (Fig. 2C and D). As we are primarily interested in the involvement of HDAC3 in transcriptional repression through histone deacetylation, a specific reduction of HDAC3 in the nucleus would be expected to be sufficient to mimic inhibition of HDAC3 histone deacetylase activity and explore its potential benefit in HD.

### Genetic reduction of *Hdac3* does not modify the R6/2 phenotype

A set of previously established quantitative tests was used to evaluate whether a genetic reduction of *Hdac3* had an effect on HD-related phenotypes in R6/2 mice. In order to generate mice for this analysis, R6/2 males were bred with *Hdac3* ^+/−^ females to generate at least 12 mice per genotype (males and females, WT n = 16, *Hdac3^+/−^* n = 14, R6/2 n = 12, Dbl n = 15). The mice were born over a period of 5 days and the 4 genotypes were recovered in a Mendelian ratio. The average CAG repeat size did not differ between the R6/2 group and the Dbl group (204±3.16 vs 205±0.91; p = 0.11). Body weight, RotaRod performance, grip strength and exploratory activity were monitored from 4 to 15 weeks of age, and in each case, a specific test was performed by the same operator, on the same day and at the same time during the weeks in which measurements were taken.

**Figure 3:**
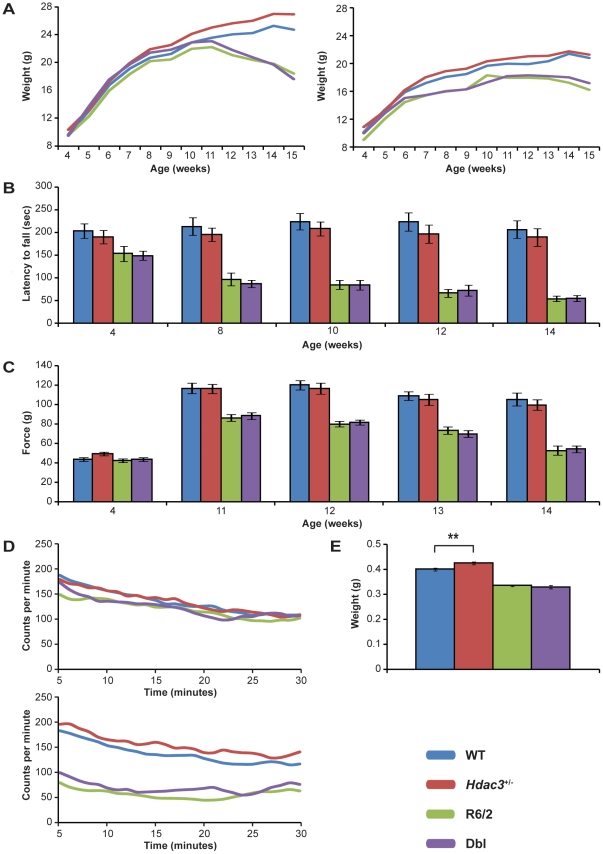
*Hdac3* genetic reduction does not modify R6/2 phenotypes. (A) Weight loss in males (left panel) and females (right panel) are shown between 4 and 15 weeks of age. *Hdac3* genetic reduction did not induce a significant increase of the body weight in R6/2 (B) RotaRod performance is represented as the average latency to fall in each group at 4, 8, 10, 12 and 14 weeks. *Hdac3* genetic reduction did not ameliorate the impairment in RotaRod performance in R6/2 (C) Average grip strength in each group is represented at 4, 11, 12, 13 and 14 weeks. *Hdac3* genetic reduction did not induce a significant improvement in the grip strength in R6/2 mice (D) Average activity for each genotype is shown at 5 (upper panel) and 13 (lower panel) weeks of age. *Hdac3* genetic reduction did not reverse the hypoactivity observed in R6/2 mice (E) Average brain weight for each group was measured at 15 weeks of age. *Hdac3* genetic reduction did not modify the brain weight loss in R6/2 but a slight increase in brain weight was observed in WT animals **p<0.01. Error bars correspond to SEM (n>12). The same color code (blue = WT; red = Hdac3; green = R6/2 and purple = Dbl) was used for all measured parameters.

Mice were weighed weekly from 4 to 15 weeks of age. As expected, R6/2 mice weighed less overall than WT mice [F_(1,49)_ = 32.657, *p*<0.001] and gained weight at a slower rate [F_(4,539)_ = 38.086, *p*<0.001] (Fig. 3A). No significant differences in the overall weight [F_(1,49)_ = 3.267, *p* = 0.077] or in weight gain [F_(4,539)_ = 0.254, *p* = 0.887] between WT and *Hdac3^+/−^* mice were observed. However, there was a trend for *Hdac*3^+/−^ mice to weigh more than WT (*p* = 0.077). Genetic reduction of *Hdac3* had no effect on R6/2 weight [F_(1,49)_ = 0.218, *p* = 0.643] and did not attenuate the rate of R6/2 weight loss [F_(4,539)_ = 1.458, *p* = 0.222]. Therefore, genetic reduction of *Hdac3^+/−^* does not improve the weight loss phenotype in the R6/2 mice.

RotaRod performance is a sensitive indicator of balance and motor coordination, which has been reliably shown to decline in R6/2 mice. RotaRod was assessed at 4, 8, 10, 12 and 14 weeks of age. Consistent with previous data, R6/2 mice displayed a significant decline in overall RotaRod performance [F_(1,49)_ = 75.523, *p*<0.001] and this worsened with age [F_(3,980)_ = 23.764, *p*<0.001] (Fig. 3B). *Hdac3* knock-down had no effect on overall RotaRod performance of *Hdac3* ^+/−^ mice [F_(1,49)_ = 0.386, *p* = 0.537] and did not significantly change the overall performance of R6/2 mice [F_(1,49)_ = 0.478, *p* = 0.493]. Furthermore, *Hdac3* knock-down had no effect on the deterioration of R6/2 RotaRod performance with age [F_(3,980)_ = 0.344, *p* = 0.792].

Forelimb grip strength was assessed at 4 weeks of age and then weekly when the mice were 11 to 14 weeks old. Consistent with previous data, R6/2 mice performance was significantly decreased compared to WT [F_(1,49)_ = 105.705, *p*<0.001] and deteriorated with age [F_(3,392)_ = 25.804, *p*<0.001] (Fig. 3C). The grip strength of *Hdac3* ^+/−^ mice was comparable to WT mice, both overall [F_(1,49)_ = 0.230, *p* = 0.634] and over the course of the experiment [F_(3,392)_ = 0.675, *p* = 0.586]. Furthermore, genetic reduction of *Hdac3* did not improve R6/2 overall grip strength performance [F_(1,49)_ = 0.603, *p* = 0.441] or deterioration with age [F_(3,392)_ = 0.846, *p* = 0.483].

Exploratory activity was assessed fortnightly from 5 to 13 weeks of age as described previously and analyzed by repeated measures general linear model (GLM) ANOVA. Mice were assessed for a period of 30 min for total activity, rearing, centre rearing and mobility. The *p*-values obtained for each parameter are displayed in Table S1. For all genotypes, the mice are highly active and mobile during the first part of the assessment period and this decreased over the course of the 30 min (Fig. 3D and Fig. S3). R6/2 mice show an overall hypoactivity and decreased mobility relative to WT mice from 7 weeks onwards. Rearing and centre rearing are also significantly decreased in R6/2 mice from 9 and 11 weeks of age respectively. *Hdac3^+/−^* mice were indistinguishable from WT mice for all of the parameters. Furthermore, *Hdac3* genetic reduction does not improve hypoactivity in the R6/2 mice.

Finally, we evaluated whether *Hdac3* genetic reduction had an effect on brain weight. Loss of brain weight has been observed in R6/2 mice in previous studies [6]. Brains were harvested from mice at 15 weeks of age, at the end of the trial. As expected, R6/2 mice displayed a significant decrease in brain weight [F_(1,49)_ = 182.333, *p*<0.001] (Fig. 3E). ANOVA analysis failed to detect any significant change in brain weight as a result of *Hdac3* genetic reduction [F_(1,49)_ = 1.865, *p* = 0.178]. Analysis of the results by Student's *t*-test revealed that *Hdac3* knock-down induces a significant increase in brain weight in the WT context (*p* = 0.0074) but no significant change was observed in the transgenic context (Fig. 3E). The brain weight of the Dbl mice is not significantly different from R6/2 (*p* = 0.3857). This result suggests that *Hdac3* knock-down has no effect on R6/2 mice brain weight loss.

### *Hdac3* genetic reduction does not ameliorate the dysregulated expression of genes of interest in R6/2 mouse brains

**Figure 4:**
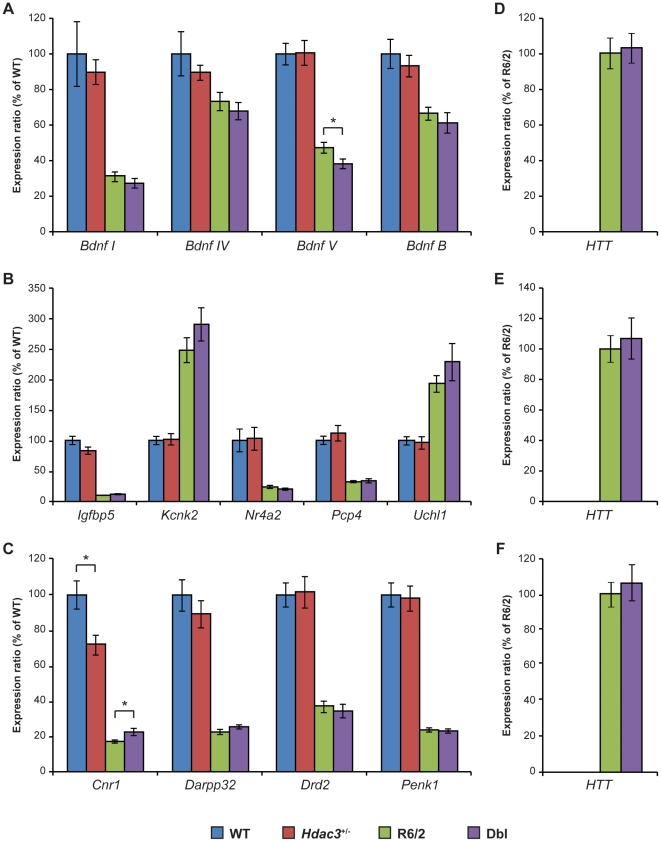
*Hdac3* genetic reduction does not reverse transcriptional dysregulation in R6/2. (A) Expression of *Bdnf* transcripts from different promoters (*Bdnf I*, *IV* and *V*) and the coding region (*Bdnf B*) in the cortex are represented as a percent of WT expression levels. With the exception of a slight decrease in *Bdnf* V, *Hdac3* reduction did not affect *Bdnf* expression. (B) Expression levels of genes specifically altered in the cerebellum of R6/2 mice are represented as a percent of WT expression. No significant difference was induced by *Hdac3* genetic reduction. (C) Expression levels of genes specifically altered in the striatum of R6/2 mice are represented as a percent of WT expression. A significant decrease in the expression of *Cnr1* in non-transgenic animals was observed as well as a slightly significant increase in *Cnr1* expression in R6/2 striata. Expression of the R6/2 transgene in Dbl brains is represented as a percent of that in R6/2 brains for cortex (D), cerebellum (E) and striatum (F). *Hdac3* reduction did not induce a significant change in transgene expression. Error bars correspond to S.E.M. (n = 8) *p<0.05. The same color code (blue = WT; red = Hdac3; green = R6/2 and purple = Dbl) was used for all the graphs. *Bdnf I, IV V*, brain derived neurotrophic factor promoter I, IV, V; *Bdnf B*, brain derived neurotrophic factor coding exon B; *Igfbp5*, insulin-like growth factor binding protein 5; *Kcnk2*, potassium channel subfamily K, member 2; *Nr4a2*, nuclear receptor subfamily 4, group A, member 2; *Pcp4*, Purkinje cell protein 4; *Uchl1*, ubiquitin C-terminal hydrolase L1; *Cnr1*, cannabinoid receptor 1; *Darpp32*, dopamine and cAMP regulated neuronal phosphoprotein; *Drd2*, dopamine D2 receptor; *Penk1*, proenkephalin.

HDAC inhibitors have been shown to reverse transcriptional dysregulation in wide variety of HD models [17], [26], [38]. In order to evaluate whether a genetic reduction of *Hdac3* might modulate the transcriptional changes observed in HD, we used RT-qPCR to analyze the expression level of a set of ‘genes of interest’ in different brain regions. This included cortical *Bdnf* as well as cerebellar and striatal genes that are dysregulated in HD mouse models [8], [12]. This analysis was performed on brain regions taken from 15 week old WT, *Hdac3^+/−^*, R6/2 and Dbl mice. Changes in gene expression induced by the transgene were consistent with previous results for all the genes that were tested (Fig. 4A, B, C). Genetic reduction of *Hdac3* had no effect on the levels of the dysregulated transcripts in R6/2 brain regions except for *Bdnf* V (decreased expression, *p* = 0.037) and *Cnr1* (increased expression, *p* = 0.028) for which very mild but statistically significant changes were observed in the cortex and in striatum respectively (Fig. 4A and C). Similarly *Hdac3* genetic reduction had no effect on the expression of these genes in non-transgenic animals except for *Cnr1* for which a significant decrease in expression was observed in *Hdac3^+/−^* compared to WT striata (p = 0.012; Fig. 4C). We also found that *Hdac3* reduction does not affect the expression of the R6/2 transgene in the cortex (Fig. 4D), cerebellum (Fig. 4E) and striatum (Fig. 4F).

### *Hdac3* genetic reduction does not reduce Huntingtin aggregation in R6/2 mouse brains

**Figure 5:**
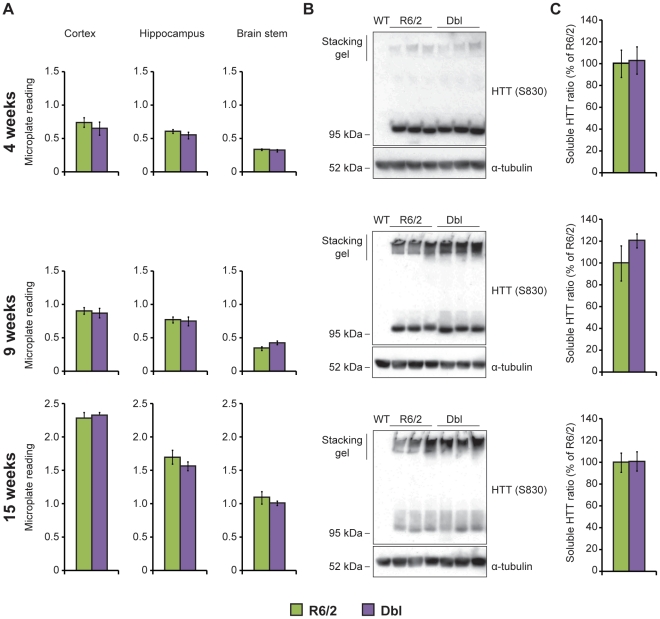
Hdac3 genetic reduction does not reduce HTT aggregation in R6/2 mouse brain. (A) The SEPRION ligand based ELISA assay was used to quantify HTT aggregation in the cortex, hippocampus and brain stem of 4, 9 and 15 week-old mice. The graphs represent the microtitre-plate reading of R6/2 (green) and Dbl (purple) lysates. Background readings obtained with WT and *Hdac3* lysates were comparable to water. Aggregation levels augment with age but are not modified by *Hdac3* reduction. Error bars correspond to S.E.M. (n>6). (B) Representative western blot of hippocampal lysates at 4, 9 and 15 weeks of age. The aggregated HTT fraction (stacking gel) augments with age whereas the soluble fraction decreases with age. α-tubulin was used as a loading control. (C) Quantification of (B). Soluble HTT is represented as a percentage of the soluble fraction in R6/2. Error bars correspond to S.E.M. (n = 6). The same color code (R6/2 = green; Dbl = purple) is used in (A) and (C).

HTT aggregation is a hallmark of brain pathology in HD and has been consistently observed in all HD models including the R6/2 mouse. Aggregation can be detected in several R6/2 brain regions from 3–4 weeks of age and increases with age [11]. In order to evaluate whether *Hdac3* reduction has an effect on this aggregation, we used a SEPRION ligand-based ELISA that provides a highly quantitative assay for measuring HTT aggregation in mouse brains [14]. Aggregates were captured from proteins extracted from the cortex, hippocampus and brain stem of 4, 9 and 15 week-old mice and detected with the MW8 antibody [39]. As expected, HTT aggregation was detectable at 4 weeks in all three brain regions, increased with age and this was not modified by *Hdac3* reduction. Levels of soluble HTT in these lysates can be quantified by western blot using the HTT specific antibody S830 [40]. The soluble trans-protein migrates at around 95 kDa whereas the aggregated protein remains in the stacking gel. Fig. 5B shows the results that we obtained for hippocampi from mice of 4, 9 and 15 weeks of age. The soluble fraction decreased with age in both R6/2 and Dbl mice and did not differ significantly between these genotypes at any time point (Fig. 5C). Similarly, a qualitative difference of the aggregated fraction was not detected. Taken together, the data obtained from the ELISA and western blotting revealed that *Hdac3* genetic reduction has no effect on HTT aggregation.

## Discussion

HDAC inhibitors were first investigated as therapeutics in the context of HD to counteract transcriptional dysregulation, thought to contribute to the molecular pathogenesis of HD. They were first shown to be protective in a *Drosophila melanogaster* model [25] and following on from the demonstration that SAHA, a potent HDAC inhibitor, improves HD-related phenotypes in the R6/2 mouse, (Hockly et al., 2003), protective effects of the butyrates in HD mouse models were also reported [22], [23]. However, therapeutic doses of SAHA were not well tolerated, leading to a marked weight loss in both WT and R6/2 animal. Therefore, if HDAC inhibitors are to be further developed as therapeutics for HD, it is essential that efficacy can be uncoupled from toxicity as far as is possible. As a pan-HDAC inhibitor, SAHA targets the eleven zinc-dependant HDACs with a preference for class I HDACs (1, 2, 3 and 8) and the class IIb HDAC, HDAC6 [30]. Identifying more specific inhibitors that target the HDAC(s) responsible for the beneficial effects observed in HD mouse models might be one mechanism by which the toxic effects could be diminished. Toward this aim, we are systematically investigating the effects of genetically reducing the levels of individual HDAC enzymes in the R6/2 mouse. In this study, we focus on HDAC3 and show that the reduction of nuclear HDAC3 to approximately 60% of WT levels has no effect on HD-related behavioral and physiological phenotypes or HTT aggregation. Importantly, the expression levels of a specific set of dysregulated gene transcripts were not restored.

HDAC3 was an interesting candidate for a number of reasons. The specific HDACs involved in the therapeutic benefits observed in invertebrate models of HD have also been explored by systematic genetic invalidation-based studies. Although a direct comparison with the mammalian enzymes cannot be made, as there are only four HDACs in *Drosophila melanogaster* and eight in *Caenorhabditis elegans* [21], [31] both studies pointed to the class I HDACs as being the critical mediators of the beneficial effects. In *Drosophila melanogaster*, *rpd3* knock-down suppressed neurodegeneration and extended the survival of transgenic flies [31]. *Rpd3* is a class I HDAC that shares amino-acid sequence similarities with human *HDAC1*, *2* and *3* at 82%, 76% and 57% respectively. In *Caenorhabditis elegans*, of the eight HDAC enzymes, it was only knock-down of *Hda-3* that induced a neuroprotective effect, whereas knock-down of other HDACs, including the other class 1 orthologues *Hda-1* and *Hda-2*, exacerbated toxicity [21]. However, it was a pharmacological study in the R6/2 mouse model of HD that indicated that HDAC3 rather than the other class I HDACs might be the most relevant in a mammalian system. HDACi **4b** is one of a series of inhibitors for which the preferred cellular target has been reported to be HDAC3 [41]. HDACi **4b** was shown to improve motor performance and body weight of R6/2 mice, associated with a reversal of histone H3 hypoacetylation and the correction of specific brain mRNA levels [26]. Given that we have found that the genetic reduction of HDAC4 has beneficial effects in the R6/2 mouse (unpublished data) and that the enzyme activity of HDAC4 is dependent on its presence in the multiprotein complex that contains HDAC3 and the SMRT/N-CoR transcriptional repressors [32], HDAC3 was a therapeutic target that merited further investigation.

Although HDAC3 is the most highly expressed HDAC in the brain [29] little is known about its function in the central nervous system. Specific silencing of *Hdac3* in the hippocampus has been shown to lead to long-term memory enhancement [42]. The overexpression of *Hdac3* in rat cerebellar neurons induces neuronal death whereas suppression of its expression by shRNA protects against low-potassium-induced neuronal death [43]. Unlike HDAC1 and 2, which are predominantly nuclear proteins, HDAC3 contains a nuclear export signal and is present in both the nucleus and the cytoplasm in mammalian cells [33], [44]. Investigations into the functions of the class I HDACs have been confined to their roles in the nucleus: HDACs 1 and 2 are part of the CoREST/mSin3a/NuRD repressor complex whereas HDAC3 interacts with NCoR/SMRT to repress gene expression [44], and in cortical neurons, HDAC3 is likely to be important for SMRT nuclear retention [45]. We were surprised to find that the cytoplasmic levels of HDAC3 in the brains of *Hdac3^+/−^* heterozygous mice were equivalent to that in WT brains, whereas nuclear levels were reduced to approximately 60% of WT. The fact that cytoplasmic HDAC3 levels are maintained in the *Hdac3* ^+/−^heterozygous state suggests that cytoplasmic HDAC3 may be tightly bound in a protein complex that sequesters HDAC3 and prevents it from migrating to the nucleus.

Our inability to detect any beneficial consequences of reducing nuclear HDAC3 levels in R6/2 mice was disappointing. The fact that *Hdac3* ^−/−^ nullizygous mice die early in embryogenesis indicates that another protein cannot compensate for HDAC3 function. We might expect that reduction of nuclear HDAC3 to approximately 60% of WT levels could be similar to the level of inhibition achieved by pharmacological approaches. Therefore, the *Hdac3* ^+/−^ heterozygous mice present a suitable model with which to further investigate the therapeutic potential of reducing HDAC3 nuclear function. Our negative results would appear to contradict the report that the administration of HDACi **4b** to R6/2 mice improves transcriptional dysregulation as well as behavioural phenotypes [26]. However these previously reported beneficial effects are surprising in the light of the physical stability and metabolic issues concerning HDACi **4b** that have recently been highlighted (Beconi et al. unpublished data). Taken together, the current data raise doubts as to the validation of HDAC3 inhibition as a therapeutic target for HD.

## Materials and Methods

### Generation of an *Hdac3* null allele

The *Hdac3*-targeting vector was generated by PCR and standard cloning. The 5′ homology arm (4.7 kb), the 3′ homology arm (4.9 kb) and the knock-out region (4.5 kb) were generated by PCR using high fidelity Prime Star HS DNA polymerase (Takara) and RP23-24H23 BAC clone as a template. Aside from the homologous arms, the final vector (HighQ1B-mHdac3) also contains loxP sequences flanking the knock-out region (4.5 kb), a neomycine resistance cassette (Neo, for positive selection of the ES cells), and a diphtheria toxin gene cassette (DTA, for negative selection of the ES cells). The targeting vector was linearized with NotI and electroporated into C57BL/6NTac ES cells (an ES cell line derived from C57BL/6 Taconic mice). The cells were selected with 200 ug/ml G418 and 192 neo resistant ES clones were screened for homologous recombination by Southern blot analysis. Targeted ES cells were electroporated with the Cre-recombinase and G418 sensitive clones were picked and analyzed by Southern blot. Clones carrying a deletion of Exon 11 to 14 were identified and microinjected into the blastocysts of C57BL/6 females to generate chimeric mice. Chimeras were bred to C57BL/6 females to achieve germ-line transmission and generate heterozygotes. The deletion was confirmed at the genomic level by PCR and sequencing.

### Mouse maintenance and breeding

All animal work was approved by the King's College London Ethical Review Panel and experimental procedures were performed in accordance with the UK Home Office regulations.

Hemizygous R6/2 mice were bred and reared in our colony by backcrossing R6/2 males to (CBA×C57Bl/6) F1 (CBF) females (B6CBAF1/OlaHsd, Harlan Olac). *Hdac3^+/−^* heterozygotes originated in a C57Bl/6 background and were backcrossed once to CBF females before breeding to R6/2 males. All animals had unlimited access to water and breeding chow (Special Diet Services, Witham, UK). Housing conditions and environmental enrichment were used as previously described [46]. *Hdac3^+/−^* females were crossed to R6/2 males to generate WT, *Hdac3* ^+/−^, R6/2 and Dbl mice. All cages contained at least one mouse of each genotype and mice were additionally given mash that consist of powdered chow mixed with water from 4 to 15 weeks. Mice were subject to a 12 h light/12 h dark cycle.

### Genotyping of R6/2 and *Hdac3* ^+/−^ mice and CAG repeat sizing

R6/2 mice were genotyped by PCR of tail-tip genomic DNA. PCR was performed using 1× Thermo-start master mix (Thermo scientific), 10% DMSO, 10 ng/ul forward primer 33727 (5′-CGCAGGCTAGGGCTGTCAATCATGCT-3′), 10 ng/ul reverse primer 32252 (5′-TCATCAGCTTTTCCAGGGTCGCCAT-3′) and 100 ng genomic DNA. Amplification conditions were: 15 min at 94°C, 35× (30 s at 94°C; 30 s at 60°C; 60 s at 72°C), 10 min at 72°C. The amplicon for the R6/2 transgene is 272 bp. The CAG repeat size was determined as follow: amplification of the CAG repeat from R6/2 mouse DNA was performed with a FAM labelled forward primer (5′-GAGTCCCTCAAGTCCTTCCAGCA-3′) and reverse primer (5′-GCCCAAACTCACGGTCGGT-3′) with 0.2 mM dNTPs; 10% DMSO; AM buffer (67 mM TrisHCL pH 8.8; 16.6 mM (NH_4_)S0_4_; 2 mM MgCl_2_; 0.17 mg/ml BSA) and 0.5 U AmpliTaq DNA polymerase (Applied Biosystems). Amplification conditions were: 90 s at 94°C, 24× (30 s at 94°C; 30 s at 65°C; 90 s at 72°C), 10 min at 72°C. All instruments and materials were obtained from Applied Biosystems unless indicated. The FAM-tagged PCR product (1 ul) together with MegaBACETM ET900 (AmershamBioscience) internal size standard (0.04 ul) were denatured for 5 min at 94°C in 9 ul of HiDi-formamide and analysed using an ABI3730 sequencer. Data analysis was performed using platemanager application GeneMapper v5.2- 3730XL. *Hdac3* ^+/−^ mice were genotyped by duplex PCR in a 10 ul reaction containing 100 ug gDNA, 2 ul of 5× Promega buffer, 1.2 ul of 25 mM MgCl2, 1 ul of 10× PCR Enhancer (Invitrogen), 1 ul of 2 mM dNTP, 1 ul of 10 uM forward 1 (F1) primer (5′- GCTTAGCCTACTTGGCAAGTGCCAG-3′), 1 ul of 10 uM forward 2 (F2) primer (5′-GGCCAGAAGCACCCAATGAGTTCTA-3′), 1 ul of 10 uM reverse (R) primer (5′- ACAATCATCAGGCCGTGAGAGTTTG-3′), 0.2 ul of Promega Taq DNA polymerase and 4.4 ul H_2_O. Amplification conditions were: 10 min at 94°C, 40× (30 s at 94°C; 30 s at 58°C; 60 s at 72°C) 10 min at 72°C. The WT allele product is 250 bp and the *Hdac3* knock-out allele is 500 bp.

### Phenotypic analysis

The phenotypic analysis was performed on mice from 4 weeks to 15 weeks of age (WT males n = 9, females n = 7; *Hdac3^+/−^* males n = 7, females n = 7; R6/2 males n = 5, females n = 7; Dbl males n = 10, females n = 5). Mice were weaned 4 days before the beginning of the analysis. Mice were weighed weekly to the nearest 0.1 g. Motor coordination was assessed using an Ugo Basile 7650 accelerating RotaRod with an acceleration set to run from 4 to 40 RPM in 300 seconds (Linton Instrumentation, UK). At 4 weeks of age, mice were tested on four consecutive days, with three trials per day. At 8, 10, 12 and 14 weeks of age, mice were tested on three consecutive days with three trials per day. Forelimb grip strength was measured once a week at 4 and from 11 to 14 weeks using a San Diego Instruments Grip Strength Meter (San Diego, CA, USA) as described in [24]. Exploratory spontaneous motor activity was recorded and assessed fortnightly at 5, 7, 9, 11 and 13 weeks of age for 30 min during the day using infra-red activity monitoring cages (Linton Instruments, AM1053) as described in [47]. Briefly, activity (total number of beam breaks in the lower level), mobility (at least two consecutive beam breaks in the lower level) and rearing (number of rearing beam breaks) were measured. The data were collected and analyzed as described in [47]. At 15 weeks of age mice were sacrificed, brains were extracted and weighed to the nearest 0.01 g.

### RNA extraction and gene expression analysis

Brain regions were dissected, snap-frozen in liquid nitrogen and stored at −80°C until required. Cortices, cerebella and striata were homogenized in Qiazol reagent and extracted using Qiagen RNeasy Mini kit (Qiagen). After quantification with a Nanodrop 1000 spectrophotometer (Labtech international), 1 ug of total RNA was reverse transcribed in 20 ul RT reaction using MMLV-RT kit (Invitrogen) and P21 random hexamers as described in Benn et al, 2008. RT reaction was diluted 10 times in nuclease-free Sigma water and 5 ul was used in 25 ul reaction containing Precision MasterMix (PrimerDesign), 300 nM gene-specific primers and 200 nM fluorescent reporting probe (FAM labelled and TAMRA quenched) specific to each target gene of interest using the Opticon2 Real Time PCR detection system (MJ research). Housekeeping genes previously identified [48] were used as ready-mixed PerfectProbe sets containing primers and probe (PrimerDesign). The geometric mean of three housekeeping genes for each brain region (*Atp5b*, *Canx*, and *Ubc* for cortex; *Atp5b*, *Eif4a2*, and *Ubc* for cerebellum, *Atp5b*, *Ubc* and *Ywhaz* for striatum) was used as a reference in order to determine relative expression ratio of the genes of interest using 2^−ΔΔCt^ method. For *Hdac3* expression analysis, primers and probe were designed using Primer3 software at the junction between exon 14 and 15 so only the WT allele can be detected (forward primer 5′- CGACGCTGAAGAGAGAGGTC -3′, reverse primer 5′- TTTCCTTGTCGTTGTCATGG-3′; probe 5′- CCGAGGAGAACTACAGCAGG-3′). The amplicon was sequence-verified. For the other target genes, primers and probes were used as in previous studies [28], [48], [49] and their sequences are available in Table S2.

### Isolation of nuclear and cytoplasmic fractions from mouse brains

Brains were extracted from mice, snap-frozen in −50°C isopenthane and stored at −80°C until required. All procedures were performed on ice. Each brain was homogenized in 2 ml ice-cold buffer 1 (575 mM sucrose; 25 mM KCl; 50 mM Triethanolamine; 5 mM MgCl2; 5 mM DTT; 0.5 mM PMSF; 1 tablet complete protease inhibitor cocktail from Roche) with 5–10 strokes in a 5 ml ice-cold homogenizer. The homogenate was then centrifuged at 800 g for 15 min at 4°C. The supernatant corresponds to the cytoplasmic fraction. The pellet was then homogenized in 3 ml buffer 1 and 2 volumes (6 ml) of Buffer 2 (2.3 M sucrose; 24.8 mM KCl; 50 mM Triethanolamine; 5 mM MgCl2; 1 mM DTT; 0.5 mM PMSF; 1 tablet complete protease inhibitor cocktail from Roche) were added and mixed to the homogenate. The mix was then centrifuged at 124000 g for 1 hour at 4°C (SW40 rotor in Beckman ultracentrifuge). The pellet (nuclear fraction) was washed twice in Buffer 1 and finally resuspended in 100 ul of Buffer 1.

### Seprion ligand ELISA assay

Brain regions were dissected, snap-frozen in liquid nitrogen and stored at −80°C until required. A 2.5% lysate was prepared by ribolysing tissues for 3×30 sec in Lysing matrix tubes (Lysing matrix D; MP Biomedicals) in ice-cold RIPA buffer (50 mM Tris-HCl pH 8.0; 120 mM NaCl; 1% Igepal; 3.125% sodium deoxycholate; 0.01% SDS; 1 mM β-mercaptoethanol; 1 uM PMSF; 1 mM DTT; 1 tablet complete protease inhibitor cocktail from Roche). Lysates were then stored at −80°C and used within 12 h. Fifteen microliters of homogenate was mixed to 3 ul 10% SDS, 62 ul H_2_O and 20 ul 5× capture buffer (Microsens Biotechnologies). A total of 100 ul was transferred to the well of a Seprion ligand-coated ELISA plate, and incubated with shaking for 1 h at room temperature (RT). The well was then washed 5× in PBS-T (PBS; 0.1% Tween) and incubated shaking for 1 h at RT with 100 ul MW8 primary antibody (mouse monoclonal antibody [39]) diluted 1∶3000 in conjugate buffer (150 mM NaCl; 4% BSA; 1% non-fat dried milk; 0.1% Tween 20 in PBS). After 5 washes with PBS-T, the well was incubated shaking for 45 min at RT with 100 ul horse radish peroxidase (HRP)-conjugated anti-mouse secondary antibody (DAKO) diluted 1∶2000 in conjugate buffer. The well was then washed 5× in PBS-T and 100 ul of RT TMB substrate (SerTec) was added and incubated in the dark at RT for 5 min. Reactions were terminated by adding 100 ul 0.5 M HCl and the absorption at 450 nm was measured using a plate reader (Biorad).

### Western blotting and antibodies

The protein concentration of the whole brain cytoplasmic fraction and RIPA extracted brain regions (ELISA) were determined using the BCA assay kit (Thermo scientific). Twenty micrograms of proteins were denatured in one volume of 2× sample buffer (125 mM Tris-HCl pH 6.8, 4% SDS, 20% Glycerol, 10% β-mercaptoethanol, 0.004% bromophenol blue) for 10 min at 95°C. Four microliters of the total 100 ul nuclear fraction was also denatured in one volume of 2× sample buffer for 10 min at 95°C. Lysates were then fractionated on 12% (nuclear and cytoplasmic fraction) or 10% (RIPA-extracted protein lysates) SDS-PAGE gels and electro-transferred into nitrocellulose membranes (Whatman) by submerged transfer apparatus (Bio-Rad) in (25 mM Tris, 192 mM glycine, 20% v/v methanol). Membranes were blocked for 1 h at RT in 5% non-fat dried milk in PBS-T (PBS; 0.2% Tween). Membranes containing nuclear and cytoplasmic fractions were then incubated with gentle agitation over night at 4°C with an HDAC3 antibody (rabbit polyclonal Abcam, 1/1000) or 20 min RT with a mouse monoclonal α-tubulin antibody (1/30000, Sigma) or rabbit polyclonal histone H4 antibody (1/5000, Millipore) in PBS-T, 5% non-fat dried milk. Membranes containing RIPA-extracted protein lysates were incubated 1 h RT with the sheep polyclonal S830 huntingtin antibody [40] diluted 1∶2000 in PBS-T, 5% non-fat dried milk or α-tubulin antibody (20 min). For chemiluminescent detection, membranes were washed 3× in PBS-T probed with HRP-linked secondary antibodies (HRP conjugated anti-rabbit mouse or sheep antibody (1∶10000, Dako) in PBS-T, 5% non-fat dried milk for 45 min at RT and washed 3× in PBS-T. Protein was detected by chemiluminescense (ECL reagent GE healthcare) according to the manufacturer's instructions. The signals were quantified using a GS-800 calibrated densitometer (Bio-Rad).

### Statistical analysis

Statistical analysis was performed by Student's *t*-test (Excel or SPSS), one-way ANOVA, two-way ANOVA and repeated measures GLM ANOVA, with the Greenhouse–Geisser correction for non-sphericity using SPSS.

## Supporting Information

Figure S1 ***Hdac3*** **genetic reduction does not affect the expression of the other** ***Hdacs*** **.** Expression of *Hdac1-11* transcripts are represented as a percent of WT expression levels in the cortex (A), the cerebellum (B) and the striatum (C) of 6 week old WT and *Hdac3* ^+/−^ mice. The level of *Hdac3* is the only significant difference between WT and *Hdac3* ^+/−^ brain regions. Error bars correspond to S.E.M. (n = 6) ***p<0.001. The same color code (blue = WT; red = Hdac3) was used for all the graphs.
(TIF)

Figure S2 **HDAC3 protein expression in** ***Hdac3+/−*** **heterozygous mouse brain.** (A) Representative western blot showing the expression of the HDAC3 protein in 4 week old mouse whole brains extracted with RIPA buffer. α-tubulin was used as a loading control (B) Quantification of (A). A slight significant decrease (≈20%) was induced by *Hdac3* genetic reduction. Error bars correspond to S.E.M. (n = 3) *p<0.05. Blue = WT; red = Hdac3.
(TIF)

Figure S3 ***Hdac3*** **genetic reduction does not modify R6/2 exploratory activity.** Average activity (left) rearing (middle) and mobility (right) for each genotype is shown at 5, 7, 9, 11 and 13 weeks of age. The same color code (blue = WT; red = *Hdac3^+/−^*; green = R6/2 and purple = Dbl) was used for all the graphs. R6/2 mice show an overall hypoactivity and decreased mobility relative to WT mice from 7 weeks onwards and rearing is significantly decreased in R6/2 mice from 9 weeks of age. *Hdac3^+/−^* mice were indistinguishable from WT mice for all of the parameters. Genetic reduction of *Hdac3* failed to induce any improvement for these parameters in R6/2 mice.
(TIF)

Table S1 ***Hdac3*** **genetic reduction does not modify R6/2 exploratory activity.** The numbers displayed in Table S1 indicate the *p*-values for each of the parameters analysed (R6/2 genotype, *Hdac3* genotype, time in the activity cages) at 5, 7, 9, 11 and 13 weeks of age. Significant *p*-values are highlighted in yellow for *p*<0.05, orange for *p*<0.01 and pink for *p*<0.001. Mice in activity cages are shown to behave differently with respect to measures of exploratory behaviour (as determined by measuring activity, mobility and rearing) over a period of 30 min (Time). R6/2 mice exhibit an overall hypoactivity and a reduced mobility from 7 weeks and rearing is significantly reduced from 9 weeks (R6/2 genotype) and (Time*R6/2 genotype). *Hdac3* genetic reduction does not influence the behaviour of mice for any parameter assessed (*Hdac3* genotype) and (Time*Hdac3 genotype) and furthermore, there appears to be no interaction between *Hdac3* and R6/2 genotypes (R6/2*Hdac3) and (R6/2*Hdac3*time).
(DOCX)

Table S2 **Sequences of primers and Taqman probes used in real-time PCR assays.** *Bdnf I, IV V*, brain derived neurotrophic factor promoter I, IV, V; *Bdnf B*, brain derived neurotrophic factor coding exon B; *Cnr1*, cannabinoid receptor 1; *Darpp32*, dopamine and cAMP regulated neuronal phosphoprotein; *Drd2*, dopamine D2 receptor; *Hdac1-11*, histone deacetylase 1–11; *Htt*, huntingtin gene; *Igfbp5*, insulin-like growth factor binding protein 5; *Kcnk2*, potassium channel subfamily K, member 2; *Nr4a2*, nuclear receptor subfamily 4, group A, member 2; *Pcp4*, Purkinje cell protein 4; *Penk1*, proenkephalin; *Uchl1*, ubiquitin C-terminal hydrolase L1.
(DOCX)
