# Supplementary figures and images for: Genetic Knock-Down of Hdac3 Does Not Modify Disease-Related Phenotypes in a Mouse Model of Huntington's Disease
Source: PLoS One. 2012 Feb 8;7(2):e31080. doi: 10.1371/journal.pone.0031080 (PMC3275566; doi:10.1371/journal.pone.0031080)

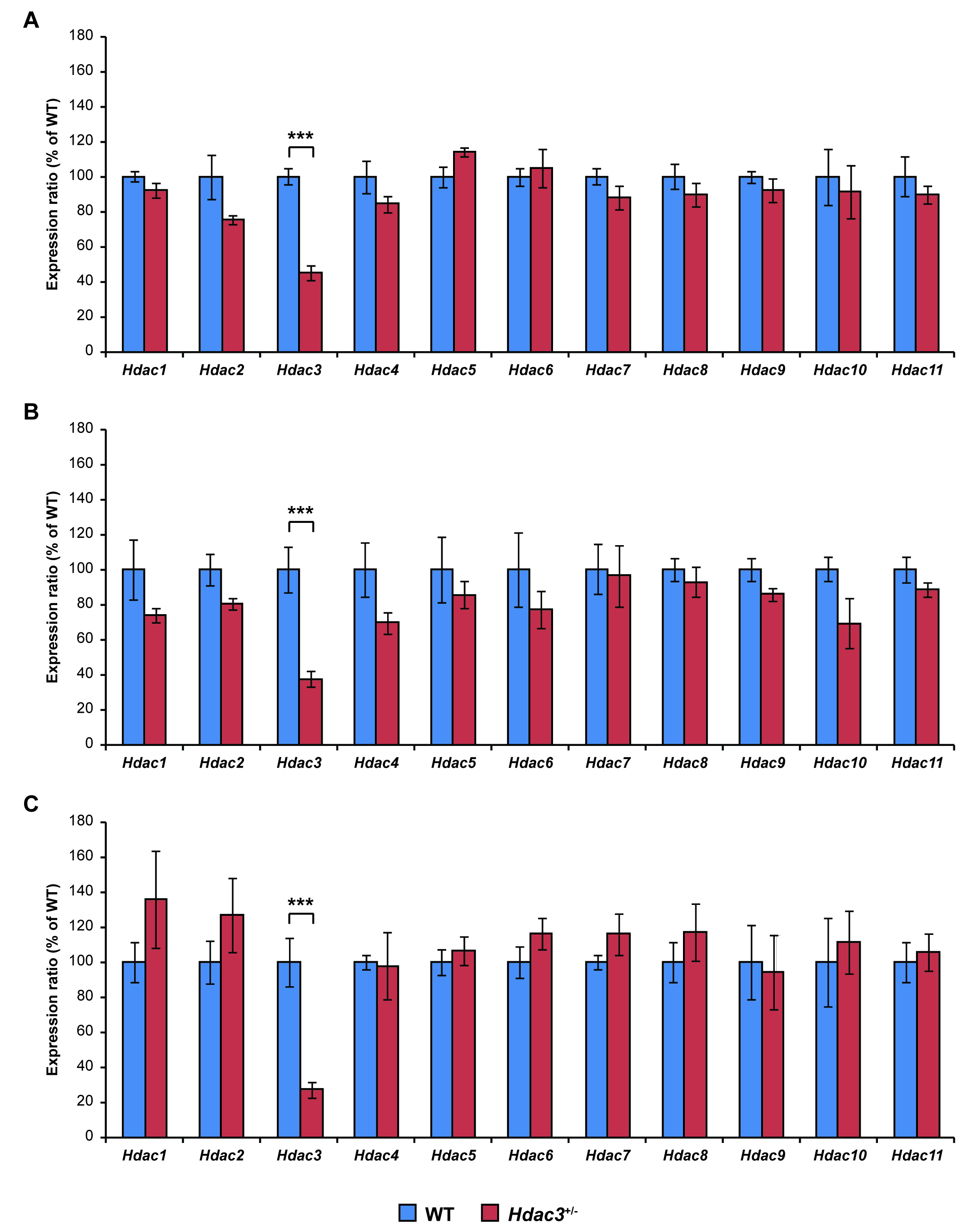

Supplement: Figure S1 — Hdac3 genetic reduction does not affect the expression of the other Hdacs . Expression of Hdac1-11 transcripts are represented as a percent of WT expression levels in the cortex (A), the cerebellum (B) and the striatum (C) of 6 week old WT and Hdac3 +/− mice. The level of Hdac3 is the only significant difference between WT and Hdac3 +/− brain regions. Error bars correspond to S.E.M. (n = 6) ***p<0.001. The same color code (blue = WT; red = Hdac3) was used for all the graphs. (TIF) [file pone.0031080.s001.tif]

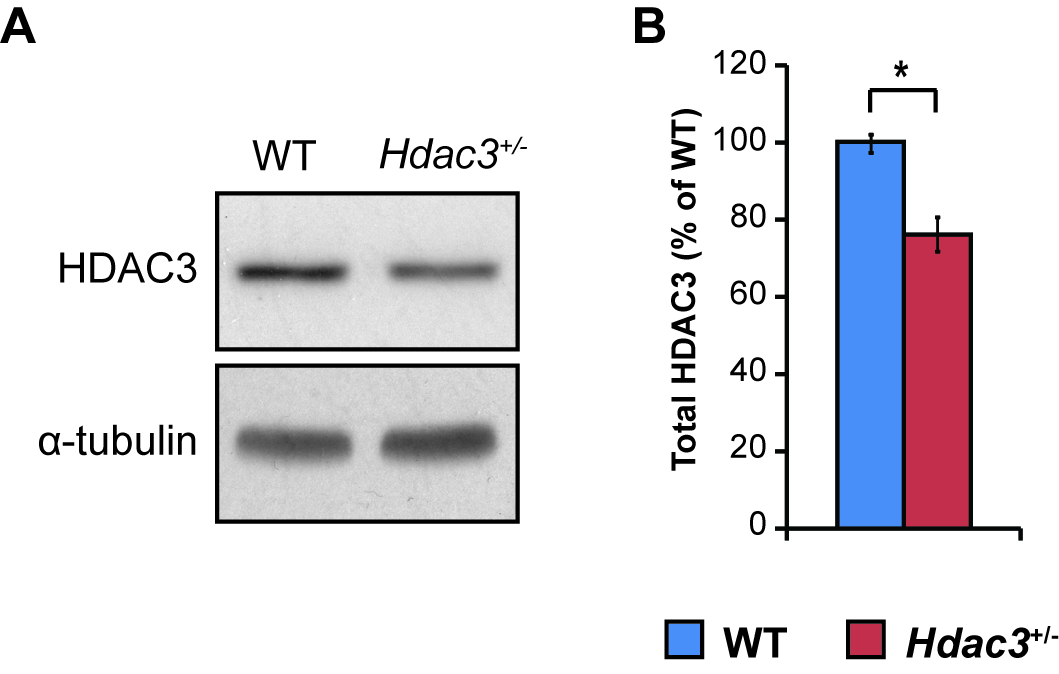

Supplement: Figure S2 — HDAC3 protein expression in Hdac3+/− heterozygous mouse brain. (A) Representative western blot showing the expression of the HDAC3 protein in 4 week old mouse whole brains extracted with RIPA buffer. α-tubulin was used as a loading control (B) Quantification of (A). A slight significant decrease (≈20%) was induced by Hdac3 genetic reduction. Error bars correspond to S.E.M. (n = 3) *p<0.05. Blue = WT; red = Hdac3. (TIF) [file pone.0031080.s002.tif]

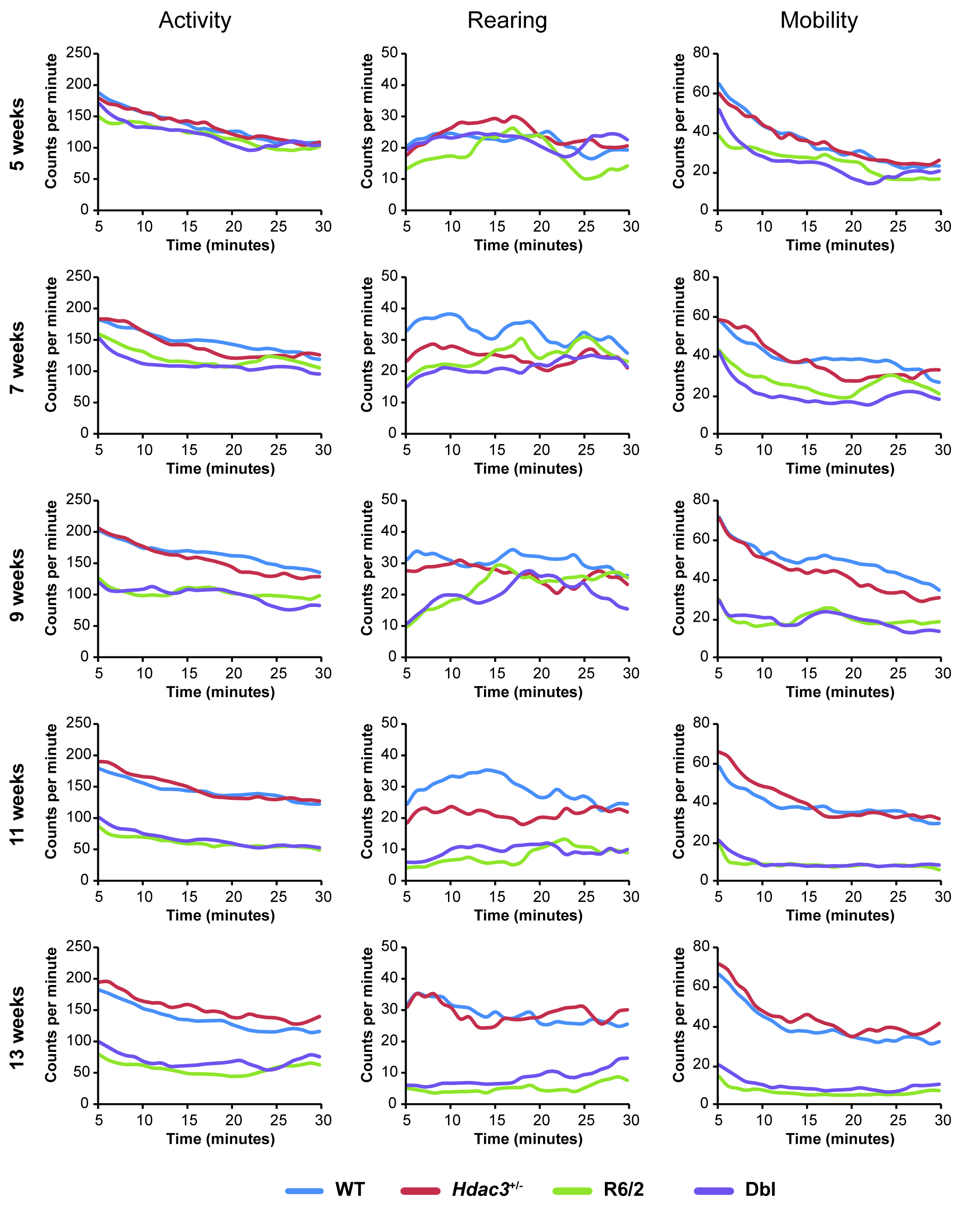

Supplement: Figure S3 — Hdac3 genetic reduction does not modify R6/2 exploratory activity. Average activity (left) rearing (middle) and mobility (right) for each genotype is shown at 5, 7, 9, 11 and 13 weeks of age. The same color code (blue = WT; red = Hdac3+/−; green = R6/2 and purple = Dbl) was used for all the graphs. R6/2 mice show an overall hypoactivity and decreased mobility relative to WT mice from 7 weeks onwards and rearing is significantly decreased in R6/2 mice from 9 weeks of age. Hdac3+/− mice were indistinguishable from WT mice for all of the parameters. Genetic reduction of Hdac3 failed to induce any improvement for these parameters in R6/2 mice. (TIF) [file pone.0031080.s003.tif]
