# Supplementary material for: Genetic Knock-Down of Hdac3 Does Not Modify Disease-Related Phenotypes in a Mouse Model of Huntington's Disease
Source: PLoS One. 2012 Feb 8;7(2):e31080. doi: 10.1371/journal.pone.0031080 (PMC3275566; doi:10.1371/journal.pone.0031080)
Supplement: Table S1 — Hdac3 genetic reduction does not modify R6/2 exploratory activity. The numbers displayed in Table S1 indicate the p-values for each of the parameters analysed (R6/2 genotype, Hdac3 genotype, time in the activity cages) at 5, 7, 9, 11 and 13 weeks of age. Significant p-values are highlighted in yellow for p<0.05, orange for p<0.01 and pink for p<0.001. Mice in activity cages are shown to behave differently with respect to measures of exploratory behaviour (as determined by measuring activity, mobility and rearing) over a period of 30 min (Time). R6/2 mice exhibit an overall hypoactivity and a reduced mobility from 7 weeks and rearing is significantly reduced from 9 weeks (R6/2 genotype) and (Time*R6/2 genotype). Hdac3 genetic reduction does not influence the behaviour of mice for any parameter assessed (Hdac3 genotype) and (Time*Hdac3 genotype) and furthermore, there appears to be no interaction between Hdac3 and R6/2 genotypes (R6/2*Hdac3) and (R6/2*Hdac3*time). (DOCX) [file pone.0031080.s004.docx]

|  | **Week** | **Activity** | **Rearing** | **Mobility** |  |  |
| --- | --- | --- | --- | --- | --- | --- |
|  | **5** | < 0.001 | 0.001 | < 0.001 |  |  |
|  | **7** | < 0.001 | 0.045 | < 0.001 |  |  |
| **Time** | **9** | < 0.001 | 0.084 | < 0.001 |  |  |
|  | **11** | < 0.001 | 0.210 | < 0.001 |  |  |
|  | **13** | < 0.001 | 0.203 | < 0.001 |  |  |
|  | **5** | 0.154 | 0.344 | 0.064 |  |  |
|  | **7** | 0.004 | 0.334 | 0.003 |  |  |
| **R6/2 genotype** | **9** | < 0.001 | 0.031 | < 0.001 |  |  |
|  | **11** | < 0.001 | < 0.001 | < 0.001 |  |  |
|  | **13** | < 0.001 | < 0.001 | < 0.001 |  |  |
|  | **5** | 0.651 | 0.767 | 0.488 |  |  |
|  | **7** | 0.295 | 0.235 | 0.343 |  |  |
| **Time * R6/2 genotype** | **9** | 0.003 | 0.008 | 0.010 |  |  |
|  | **11** | 0.461 | 0.234 | 0.018 |  |  |
|  | **13** | 0.001 | 0.035 | < 0.001 |  |  |
|  | **5** | 0.715 | 0.234 | 0.737 |  |  |
|  | **7** | 0.722 | 0.420 | 0.919 |  |  |
| ***Hdac3* genotype** | **9** | 0.788 | 0.514 | 0.800 |  |  |
|  | **11** | 0.406 | 0.743 | 0.475 |  |  |
|  | **13** | 0.312 | 0.452 | 0.359 |  |  |
|  | **5** | 0.652 | 0.166 | 0.390 |  |  |
|  | **7** | 0.893 | 0.626 | 0.357 |  |  |
| **Time * Hdac3 genotype** | **9** | 0.384 | 0.397 | 0.831 |  |  |
|  | **11** | 0.592 | 0.778 | 0.196 |  |  |
|  | **13** | 0.440 | 0.156 | 0.870 |  |  |
|  | **5** | 0.849 | 0.535 | 0.969 |  |  |
|  | **7** | 0.805 | 0.662 | 0.709 |  |  |
| **R6/2 * *Hdac3* geno.** | **9** | 0.585 | 0.875 | 0.489 |  |  |
|  | **11** | 0.728 | 0.201 | 0.982 |  |  |
|  | **13** | 0.923 | 0.832 | 0.876 |  |  |
|  | **5** | 0.303 | 0.371 | 0.170 |  |  |
|  | **7** | 0.467 | 0.648 | 0.536 |  |  |
| **Time * R6/2 * *Hdac3* geno.** | **9** | 0.308 | 0.225 | 0.547 |  |  |
|  | **11** | 0.749 | 0.220 | 0.767 |  |  |
|  | **13** | 0.332 | 0.532 | 0.914 |  |  |
|  |  |  |  |  | |  |
|  | *p*<0.001 | *p*<0.01 | *p*<0.05 |  | |  |
