# Supplementary material for: Genetic Knock-Down of Hdac3 Does Not Modify Disease-Related Phenotypes in a Mouse Model of Huntington's Disease
Source: PLoS One. 2012 Feb 8;7(2):e31080. doi: 10.1371/journal.pone.0031080 (PMC3275566; doi:10.1371/journal.pone.0031080)
Supplement: Table S2 — Sequences of primers and Taqman probes used in real-time PCR assays. Bdnf I, IV V, brain derived neurotrophic factor promoter I, IV, V; Bdnf B, brain derived neurotrophic factor coding exon B; Cnr1, cannabinoid receptor 1; Darpp32, dopamine and cAMP regulated neuronal phosphoprotein; Drd2, dopamine D2 receptor; Hdac1-11, histone deacetylase 1–11; Htt, huntingtin gene; Igfbp5, insulin-like growth factor binding protein 5; Kcnk2, potassium channel subfamily K, member 2; Nr4a2, nuclear receptor subfamily 4, group A, member 2; Pcp4, Purkinje cell protein 4; Penk1, proenkephalin; Uchl1, ubiquitin C-terminal hydrolase L1. (DOCX) [file pone.0031080.s005.docx]

| **GENE** | **FORWARD PRIMER** | **REVERSE PRIMER** | **PROBE (5’ 6-FAM; 3’ TAMRA)** |
| --- | --- | --- | --- |
| ***Bdnf I*** | GCAAAGCCGAACTTCTCACAT | GCAACCGAAGTATGAAATAACCATAG | TTCCACCAGGTGAGAAGAGTGATGACCAT |
| ***Bdnf IV*** | CTGCCTTGATGTTTACTTTGACAAG | GCAACCGAAGTATGAAATAACCATAG | TTCCACCAGGTGAGAAGAGTGATGACCAT |
| ***Bdnf V*** | GGGATCCGAGAGCTTTGTG | GCAACCGAAGTATGAAATAACCATAG | TTCCACCAGGTGAGAAGAGTGATGACCAT |
| ***Bdnf B*** | GGGTCACAGCGGCAGATAAA | GCCTTTGGATACCGGGACTT | TCTGGCGGGACGGTCACAGTCC |
| ***Cnr1*** | CACAAGCACGCCAATAACACA | ACAGTGCTCTTGATGCAGCTTTC | CAGCATGCACAGGGCCGC |
| ***Darpp32*** | CCCGACAGGTGGAGATGATC | GCTGCACAGCTTTCAGTGATG | CTGCCATGCTTTTCCGGGTCTCAG |
| ***Drd2*** | ACACCACTCAAGGGCAACTGT | GGCGGGCAGCATCCA | GGGTCAGGACATGAAACTCTGCACCG |
| ***Hdac1*** | TCTGAATACAGCAAGCAGATGCA | ACAGAACTCAAACAAGCCATCAAAC | AGATTCAATGTTGGTGAGGACTGTCCGG |
| ***Hdac2*** | AGAAGATTGTCCGGTGTTTGATG | CACAGCCCCAGCAACTGAA | TTGAGTTTTGTCAGCTCTCCACGGGTG |
| ***Hdac3*** | CGACGCTGAAGAGAGAGGTC | TTTCCTTGTCGTTGTCATGG | CCGAGGAGAACTACAGCAGG |
| ***Hdac4*** | CTGGCATCCCTGTGTCATTTG | ACACAAGACCTGTGGTGAACCTT | CTGCCACCTTCCCCATGTCAGTCC |
| ***Hdac5*** | GCAACAAGGAGAAGAGCAAAGAG | TCCTGGAGCCTCAGCTTTACC | TGCCATCGCCAGCAC |
| ***Hdac6*** | GGAGACAACCCAGTACATGAATGAA | CGGAGGACAGAGCCTGTAG | TATCTGCATCCGAACTCATATTCCTGTGCC |
| ***Hdac7*** | CCCACCTGTCAGACCCAAGT | AGTCATAGACCAGCCCTGTAGCA | CTCAACAGCTCAGAGACA |
| ***Hdac8*** | GGCCCATCCATCCCTGTAG | TTTAGATCGCCGGAGACAGTTT | TGGACGAGGGACCAGG |
| ***Hdac9*** | TGGCAGAATCCTCGGTCAGT | CCCAGCAGGGCCATTGT | TCTCCAGGGTCAGGTCCCAGTTCACC |
| ***Hdac10*** | CCGCTATGAGCATGGAAGCT | CAACTGCATCTGCATCAGACTCT | CTGGCCGTTTCTC |
| ***Hdac11*** | TGGGCATGAGCGAGACTTC | GCGGTTGTAAACATCCATGATG | TGGGTGACAAGCGAG |
| ***Htt*** | GCTGCACCGACCGAGT | CGCAGGCTGCAGTTAC | CAGCTCCCTGTCCCGGCGG |
| ***Igfbp5*** | AAGGATTCTACAAGAGAAAGCAGTGTAA | ACTTGTCCACACACCAGCAGAT | TCCCGTGGCCGCAAACGTG |
| ***Kcnk1*** | GACTACGTGGCAGGTGGATCA | GCCAGCCCAACGAGGAT | AATATCTGGACTTCTACAAGCCTGTGGTGTG |
| ***Nr4a2*** | ATTTCCTCGAAAACTCCAATAACTCT | TGAGGCGAGGACCCATACTG | CTGAAGCCATGCCTTGTGTTCAGGC |
| ***Pcp4*** | CTGAGCTGTTCTGTGGGACCTA | CGCTCCGGCACTTTGTCT | CTGCGGAGTCAGGCCAACATGA |
| ***Penk1*** | ATGCAGCTACCGCCTGGTT | GCAGCTGTCCTTCACATTCCA | AGGCGACATCAATTTCCTGGCGTG |
| ***UchL1*** | GGTACCATCGGGTTGATCCA | AACTGTTTCAGGACGGATCCA | AACCAAGACAAGCTGGAATTTGAGGA |
